# Cleavage of the IPS-1/Cardif/MAVS/VISA does not inhibit T cell-mediated elimination of hepatitis C virus non-structural 3/4A-expressing hepatocytes

**DOI:** 10.1136/gut.2007.147264

**Published:** 2008-08-19

**Authors:** G Ahlén, E Derk, M Weiland, J Jiao, N Rahbin, S Aleman, D L Peterson, K Pokrovskaja, D Grandér, L Frelin, M Sällberg

**Affiliations:** 1 Division of Clinical Microbiology, Karolinska Institutet at Karolinska University Hospital Huddinge, Stockholm, Sweden; 2 Centre for Gastroenterology, Karolinska University Hospital Solna, Sweden; 3 Commonwealth University, Richmond, Virginia, USA; 4 Department of Oncology and Pathology, Cancer Center Karolinska, Karolinska University Hospital Solna and Karolinska Institutet, Stockholm, Sweden

## Abstract

**Background:**

Hepatitis C virus (HCV) effectively establishes persistent infection in human livers. The non-structural (NS) 3/4A complex participates in this process by cleavage of interferon β (IFNβ) promoter stimulator-1 (IPS-1; also termed Cardif/MAVS/VISA), which inhibits responses to double stranded (ds) RNA. However, it is not known whether this effect extends beyond innate responses.

**Aims:**

To test if HCV NS3/4A affects innate and adaptive immune responses in vivo.

**Methods:**

NS3 levels were semi-quantified in human liver biopsies, transfected cells, and in transgenic (Tg) mouse livers by western blot. The effect of NS3/4A on dsRNA-mediated signalling and on the integrity of IPS-1 was analysed using in vitro translation, transfected cells and Tg mice. Cytotoxic T cell (CTL)-mediated clearance of transient firefly luciferase (FLuc)- and/or NS3/4A-Tg hepatocytes was determined using in vivo imaging and western blot.

**Results:**

NS3 protein levels were in a comparable range (0.1–49 μg/g tissue) in infected human livers and Tg mouse livers. Importantly, these levels of NS3/4A reduced murine innate responses to synthetic dsRNA in vivo, supporting the possibility that this occurs also in infected humans. The likely explanation for this was the NS3/4A-mediated cleavage of mouse IPS-1, albeit less efficiently than human IPS-1. Despite this, FLuc- and/or NS3/4A-expressing murine hepatocytes were effectively eliminated by hepatic CTLs, utilising the classical molecules for virus-infected cell lysis, including CD8, IFNγ, perforin and FasL.

**Conclusions:**

Although HCV NS3/4A inhibits the innate immunity, this does not prevent CTL-mediated clearance of NS3/4A-expressing hepatocytes in vivo. Thus, other HCV proteins are most likely responsible for interfering with the adaptive immunity.

Hepatitis C virus (HCV) is highly efficient in establishing persistent infections in human hepatocytes in vivo, but not in vitro.^1^ ^2^ Several HCV proteins protect infected cells against the various ways that the host can shut down or eliminate infected cells^3^ and the specific T cell responses seem to be deregulated during chronic infection.^4^^–^10 This impairment may be caused by escape mutations within T cell epitopes^11^^–^14 or by an inhibitory effect directly exerted by viral proteins.^4^ Indeed, transgenic (Tg) mice expressing the complete HCV polyprotein have an impaired clearance of hepatotropic infections,^15^ but the protein/s responsible has/have yet not been identified. Examples of events documented in transgenic mice are inhibition of Fas-mediated apoptosis, reduced sensitivity to tumour necrosis factor α (TNFα)-mediated apoptosis, and reduced interferon (IFN) responses.^15^^–^17 However, these studies are hampered in that we do not know whether similar effects can be observed in both human and murine cells.

HCV infection itself does not seem to be highly immunogenic.^18^ Certain HCV proteins, in particular the non-structural (NS) 3/4A complex, have been found to modulate the host response.^3^ The NS3/4A protease has been shown to cleave adaptor proteins transducing the host cell response to the double stranded (ds) RNA generated during replication of the viral genome.^19^ ^20^ HCV dsRNA is synthesised by the membrane-bound replication complex in the endoplasmic reticulum and lipid droplets^21^ can activate the retinoic acid-inducible gene-I (RIG-I) pathway. The RIG-I pathway activates nuclear factor κB (NF-κB) and IFN responses, which switches the cell into an antiviral state.^3^ ^22^ However, to prevent this response, the protease domain of the HCV NS3/4A complex cleaves the IFNβ promoter stimulator-1 (IPS-1; also known as caspase recruitment domain adaptor inducing IFNβ (Cardif), mitochondrial anti-viral signalling protein (MAVS) and virus-induced signalling adaptor (VISA)) signal transducer from the mitochondria. This prevents nuclear translocation of transcription factors interferon regulatory factor 3 (IRF-3) and NF-κB,^23^ and inhibits IFN responses and apoptosis. However, most of these results have been generated from transfected tumour cells in vitro, and less is known about any in vivo consequences.^3^ ^19^ ^23^ New data obtained using unrelated RNA viruses suggest that IPS-1 does not influence adaptive immune responses.^24^ ^25^ Hence, although it is clear that the NS3/4A protein in vitro inhibits innate signalling through IPS-1, it is not known if this affects the adaptive immune responses.

To better understand the in vivo role of NS3/4A-mediated inhibition of innate responses seen in human tumour cells transfected by HCV NS3/4A, we aimed to determine whether effects similar to those reported could be observed in murine cells. This is certainly of importance since this would allow detailed in vivo studies in Tg mice that mimic events taking place in the human liver.

## MATERIALS AND METHODS

### Human liver biopsies

Liver biopsies were taken from six patients undergoing clinical routine work-up using an aspirating 1.6 mm diameter needle. A 5–10 mm part of the biopsy was snap-frozen in liquid nitrogen. Five patients had confirmed chronic HCV infections with a viral load of 14.2×10^6^ (male, gt1), 8.8×10^6^ (male, gt3a), 0.45×10^6^ (male, gt3a) and 0.44×10^6^ IU/ml (female, gt1a). For one male patient infected by HCV gt1 the viral load had not been determined. All HCV-infected patients had inflammation ranging from grade I to grade III and fibrosis ranging from grade I to grade IV. A patient with a liver disease not caused by HCV was used as a negative control and had minimal inflammation and intermediate steatosis.

### Animals

Inbred C57BL/6 (H-2^b^), BALB/c (H-2^d^) (Charles River, Sulzfeld, Germany), Perforin−/− (H-2^b^), FasL−/− (H-2^b^) (The Jackson Laboratory, Bar Harbor, Maine, USA), CD4−/− (H-2^b^), CD8−/− (H-2^b^), TLR4−/− (H-2^b^) and IFNγR2−/− (H-2^b^) 4–8-week-old mice were obtained from the breeding facility at the Department of Microbiology, Tumor and Cell Biology, Karolinska Institutet, Sweden. C57BL/6 and CBAxC57BL/6 Tg mice with intra-hepatic expression of the NS3/4A proteins^17^ were bred and maintained in-house.

### Plasmid DNA, recombinant protein and synthetic peptides

Plasmids containing a full-length wild-type (wt) NS3, wtNS3/4A, mutant (mut) NS3/4A with a mutation that prevents cleavage between NS3 and NS4A, three alanine mutant NS3/4A genes where the cleavage of NS3-NS4A was retained,^13^ ^14^ and a codon optimised (co) NS3/4A gene in pVAX1 have been described.^26^^–^28 Genes coding for FLuc2- (Promega, Madison, Wisconsin, USA) and human (h) and mouse (m) IPS-1 (InvivoGen) were separately cloned into pVAX1 using standard techniques. The pISRE-TA-Luc and pRL-TA-Luc plasmids were purchased from Promega.

Recombinant NS3 (rNS3) protein has been described.^17^ The NS3-derived major histocompatibility complex (MHC) class I H-2D^b^-restricted GAVQNEVTL peptide and the HBc-derived H-2K^b^-restricted MGLKFRQL peptide were kindly provided by Dr Michael Levi, Tripep AB, Stockholm, Sweden.^17^ Peptides were synthesised using standard protocols with 9-fluorenylmethoxycarbonyl to protect the amino acids.^29^

### Cell lines

The SP2/0-Ag14 (H-2^d^), RMA-S cells (kindly provided by Professor K Kärre, Karolinska Institutet, Sweden), HepG2, and HEK293 were maintained as described.^17^ SP2/0-Ag14 cells with stable expression of NS3/4A^23^ were maintained with 800 μg/ml geneticin.

### Transient transfection and protein sample preparation

HepG2 and SP2/0 cells were transiently transfected as described.^23^ In brief, the cells were plated into 10 cm^2^ wells (5×10^6^ cells/well) the day before transfection. Two micrograms of each pVAX1 plasmid was transfected into cells using Lipofectamine 2000 reagent (Invitrogen, Carlsbad, California, USA). Cells were either co-transfected using 0.2–2 μg/μl poly(I:C) or treated with 0.2–2 μg/μl poly(I:C) 5 h after transfection or left untreated. After transfection, the cells were incubated for 16–36 h at 37°C, 5% CO_2_ prior to harvest and subsequent isolation of cytoplasmic and nuclear protein fractions, according to standard protocols. In brief, cells were re-suspended in lysis buffer (10 mmol/l HEPES–KOH, 1.5 mmol/l MgCl_2_, 10 mmol/l KCl, 0.2 mmol/l phenylmethanesulfonyl fluoride (PMSF), 1 mmol/l Na_3_VO_4_ and 0.5 mmol/l dithiothreitol (DTT)), sonicated and incubated on ice for 10 min. Then the samples were centrifuged and the supernatant containing the cytoplasmic portion was collected. The remaining cell pellet was re-suspended in a different lysis buffer (20 mmol/l HEPES–KOH, 1.5 mmol/l MgCl_2_, 420 mmol/l KCl, 0.2 mmol/l EDTA, 25% glycerol, 0.2 mmol/l PMSF, 1 mmol/l Na_3_VO_4_ and 0.5 mmol/l DTT) and incubated for 30 min on ice before the nuclear portion was collected by centrifugation. All protein extracts were stored at −80°C until analysis.

### Detection of IRF-3, NF-κB and IPS-1 by western blot

Cytoplasmic and nuclear extracts from transiently transfected cells were loaded on a 4–12% Tris–Bis gel (Invitrogen) for analysis by using sodium dodecyl sulfate polyacrylamide gel electrophoresis (SDS–PAGE). Proteins were transferred to an Invitrolon/polyvinylidene difluoride (PVDF) membrane (Invitrogen) for 1 h at 30 V. For detection of IRF-3, NF-κB and IPS-1, a rabbit-IRF-3 antibody (#4962; Cell Signaling, Danvers, Massachusetts, USA), a rabbit-NF-κB p65 antibody (#3034; Cell Signaling) or a rabbit-IPS-1 antibody (AT107; Alexis Biochemicals, San Diego, California, USA) was used at a dilution of 1:1000, 1:1000 and 1:2000, respectively. Bound antibody was detected using an enzyme conjugated goat-anti-rabbit antibody (Dako, Glostrup, Denmark), and proteins were visualised using the ECL Plus western blotting reagent (GE Healthcare, Uppsala, Sweden) according to manufacturer’s protocol.

### Luciferase reporter assay

A reporter plasmid containing the interferon stimulatory response element (ISRE) enhancer upstream of the TATA box and luciferase (Clontech, Mountain View, California, USA) were used for transient transfections of HEK293 cells. For transfections, 10^5^ cells were plated per well in a 24-well plate before co-transfected with 0.125 μg of pISRE-TA-Luc (firefly) reporter plasmid, 0.01 μg of pRL-TK (Renilla) reporter plasmid and 0.375 μg empty pVAX1 plasmid or pVAX1 plasmid containing wtNS3, wtNS3/4A or coNS3/4A using the Lipofectamine 2000 reagent (Invitrogen). At 16 h post-transfection the cells were treated with 2 μg/μl poly(I:C). Cell lysates collected 6–24 h after poly(I:C) treatment were analysed for luciferase activity using the dual-luciferase reporter assay system (Promega) according to the manufacturer’s protocol. Light emission was detected in an Anthos Lucy2 luminometer and was analysed using the Anthos LucySoft software (Anthos Labtech Instruments, Vienna, Austria) and Microsoft Excel.

### In vitro transcription and translation assay

The cleavage of mIPS-1 was analysed by co-expression of 1 μg mIPS-1 and 1 μg wtNS3, wtNS3/4A or coNS3/4A DNA plasmids by an in vitro transcription and translation assay (TNT; Promega), as described.^14^ ^26^

### Immunisation and treatment protocols

DNA immunisations were performed by regular intramuscular (i.m.) immunisation in the tibialis anterior muscle (50 μg/dose) or by gene gun-based immunisation (2 μg/dose) exactly as described previously.^26^ ^27^

The effects of synthetic dsRNA in vivo was studied by injecting 100 μg poly(I:C) (Invitrogen) in 200 μl sterile phosphate-buffered saline (PBS) or 200 μl sterile PBS alone intravenously into the tail vein. The effect of TNFα was studied after i.p. injection of TNFα at doses of 5–20 μg/kg with d-galactosamine (d-GalN) (20 mg) in 100 μl PBS as previously described.^17^

### Determination of HCV NS3/4A-protein-expressing liver cells in vivo

NS3 protein expression was determined in human (5 mg) and murine (100 mg) livers as described.^26^ ^27^ ^30^ Groups of mice were immunised once using the gene gun or left untreated. Two weeks later the mice were given a hydrodynamic injection of the coNS3/4A plasmid, exactly as described previously.^30^ ^31^ The presence of NS3/4A-protein expression was determined 72 h later by immuno-precipitation and western blot as described previously.^26^ ^27^ ^30^

For in vivo imaging, groups of mice were immunised once intramuscularly in the right tibialis anterior muscle with either 50 μg of Luc2-pVAX1 plasmid, 50 μg of the coNS3/4A plasmid, or left untreated. Two weeks later the mice were given a hydrodynamic injection containing 50 μg Luc2 and 50 μg coNS3/4A plasmid. The presence of reporter gene expression was determined from 7 h to 192 h by bioluminescence in anaesthetised mice using the In Vivo Imaging System 100 (IVIS 100, Xenogen, Hopkinton, Massachusetts, USA). To detect the presence of reporter gene expression the luciferin (Xenogen) substrate was injected and the reporter gene expression was analysed 5–10 min later for 1.5 and/or 60 s (depending on time point). Images and assessment of emitted light were analysed with Living Image software (version 2.50; Xenogen).

### Detection of NS3/4A-specific lytic CTLs, IFNγ-producing CTLs and T helper cells, and intra-hepatic T cells

Lytic cytotoxic T cells (CTLs) in immunised mice were detected in individual mouse spleens by a standard 4-h ^51^Cr-release assay using NS3 peptide (GAVQNEVTL) loaded H-2D^b^-expressing RMA-S target cells exactly as described previously.^30^ The presence of IFNγ-producing CTLs and T helper (Th) cells to NS3 was detected in pooled splenocyte cultures by a commercially available ELISpot assay as described previously.^13^ ^30^

The detection of CD3+ T cells in the liver was performed by immunohistochemistry as described previously.^30^ The mean values of staining scores and standard deviations were calculated for vaccinated and non-vaccinated animals and compared using the Mann–Whitney U test.

### Statistical analysis

Statistical comparisons were performed using the Statview 5.0 (3/20/98, Power PC version, SAS Institute) and Excel:mac (version 11.3.7, Microsoft) software packages for Macintosh. Parametrical data were compared using the Student t test (Statview and Excel) and non-parametric data were compared using the Mann–Whitney U test (Statview). Frequencies were compared using Fisher’s exact test (Statview).

## RESULTS

### HCV NS3/4A gt1a interferes with IFN signalling and NF-κB activation in human and murine tumour cells

**Figure 1:**
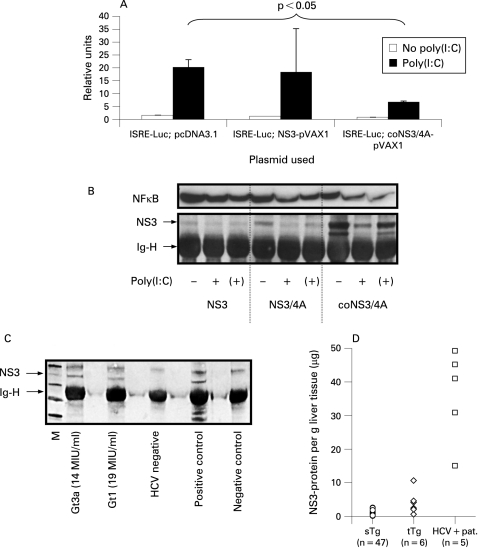
Inhibition of interferon (IFN) responses to dsRNA in human cells (A) determined by firefly luciferase (FLuc) expression in cell lysates of HEK293 cells 36 h after co-transfection with pISRE-Luc reporter plasmid and indicated NS3- and NS3/4A-containing plasmids. Mean values were compared using the Student t test. The effect of NS3 and NS3/4A on poly(I:C)-induced nuclear factor kappaB (NF-κB) in transiently transfected SP2/0 (H-2^d^) cells (B). The “+” indicates cells co-transfected with poly(I:C), “(+)” indicates cells treated with poly(I:C) 5 h post-transfection. Detection of NS3 protein in human liver biopsies (5 mg) by immunoprecipitation and western blot analysis (C). “M” indicates the molecular rainbow marker, “Pos ctrl” NS3/4A transiently transfected HepG2 cells, “Neg ctrl” is a wild-type mouse liver (C). Semi-quantification of NS3-protein expression (μg/g liver tissue) in human livers, stable and transiently NS3/4A-transgenic mouse livers (D). All experiments were run at least three times, except for 1(D), which is a summary of data from several experiments. HCV, hepatitis C virus; ISRE, interferon stimulatory response element.

Interference of our gt 1a NS3/4A sequence with IFN responses was tested in human HEK-293 cells co-transfected with NS3 and a reporter plasmid controlled by an IFN-sensitive promoter. This revealed that synthetic dsRNA, ie, poly(I:C)-induced IFN reporter expression, was impaired by the NS3/4A genes (fig 1A). Also, the coNS3/4A gene reduced NF-κB activation in transiently transfected poly(I:C)-treated HepG2 cells (data not shown). Thus, our gt1a NS3/4A gene behaves the same way as other NS3/4A sequences.^19^ ^32^

We next tested if NS3/4A had similar effects in murine tumour cells. First, the basal level of NF-κB was reduced in SP2/0 cells stably expressing the NS3/4A-proteins (data not shown), and in transiently NS3/4A-transfected poly(I:C)-stimulated SP2/0 cells (fig 1B). No difference in MHC class I expression was seen between non-transfected and NS3/4A-transfected SP2/0 cells (data not shown). Collectively, NS3/4A affects NF-κB responses in murine tumour cells, but not class I antigen presentation.

### Expression levels of NS3-protein in human and murine livers

To explore the effects of NS3/4A on non-tumour cells in vivo, we used our different NS3/4A-Tg mouse models.^17^ ^30^ We first compared the levels of NS3 expression between human HCV infected livers and livers from the Tg mice (fig 1C,D). NS3 levels were estimated using a standard curve obtained with dilutions of recombinant NS3 (fig 1D). In liver biopsies from two patients with high viral load (>800 000 IU/ml serum) the levels of NS3 were 45 (gt1a) and 41 μg/g (gt3a) tissue, respectively (fig 1C, lanes 2 and 4, respectively). In another two patients, each with an intermediate viral load (450 000 and 550 000 IU/ml, respectively), the NS3 levels were 31 and 15 μg/g tissue, respectively (fig 1D). From one patient with an unknown viral load the NS3 levels were 49 μg/g liver tissue (fig 1D). The average NS3 expression levels in five human livers were 36 (SD 14) μg/g tissue. An analysis of the NS3 levels in stable Tg (sTg) mice ranged from 0.1 to 0.5 μg in females (n = 7; mean 0.3 (SD 0.2) μg/g) and from 0.2 to 4 in males (n = 42; mean 1.5 (SD 3.7) μg/g), consistent with previous observations.^17^ In the transiently Tg (tTg) mice (n = 6) the levels ranged from 0.5 to 11 μg/g tissue, with a mean of 4 (SD 4) μg/g tissue. Taken together, this suggests that the mean levels of NS3 in the male stable NS3/4A-Tg mice were on average 22-fold lower as compared to four patients with a viral load exceeding 400 000 IU/ml. In contrast, the NS3 levels in livers from the transient Tg mice were more within the same range as the tested human livers, on average 8-fold lower, and may possibly even exceed those in patients with a lower viral load. Thus, the NS3 levels seem to be lower in the stable Tg mice as compared to humans with a viral load exceeding 400 000 IU/ml, whereas the NS3 levels in the transiently Tg mice are more comparable to humans. Finally, a larger study on NS3 expression in human livers is under way, and we have not seen a band corresponding to NS3 in any of more than 20 patients negative for HCV infection (Rahbin *et al*, manuscript in preparation). Also, we have never seen a band corresponding to NS3 in a non-transgenic mouse liver. Thus, the specificity of the currently used western blot assay seems be satisfactory.

### The role of NS3/4A on in vivo responses to dsRNA

**Figure 2:**
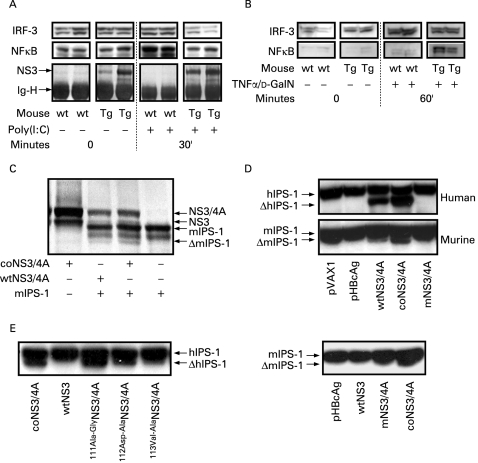
NS3/4A-protein expression in mice disturbs responses to synthetic dsRNA. The nuclear translocation of interferon regulatory factor 3 (IRF-3) and nuclear factor κB (NF-κB) by intravenous poly(I:C) is reduced in stable NS3/4A-Tg mice (A), but not by tumour necrosis factor α (TNFα)/d-GalN treatment (B), representing a retinoic acid inducible gene-I (RIG-I)-independent pathway. Cleavage of murine (m) interferon β (IFNβ) promoter stimulator-1 (IPS-1) by the NS3/4A protease was determined using a standard in vitro transcription and translation assay in presence of ^35^S-methionine and sodium dodecyl sulfate polyacrylamide gel electrophoresis (SDS–PAGE) (C). The loading of each lane is indicated under the gel. Expression of mIPS-1 alone generates a single protein band, whereas mIPS-1 co-expressed with wtNS3/4A or coNS3/4A, but not wtNS3 (data not shown), generates two distinct protein bands, one representing mIPS-1 and the other the truncated mIPS-1 (C). Detection of cleaved hIPS-1 (ΔhIPS-1) and ΔmIPS-1 in HepG2 cells (D) co-transfected with human (upper gel) or mouse (lower gel) IPS-1 by HCV NS3/4A and appropriate controls. Uncleaved IPS-1 generates one single band in the western blot analysis. In the presence of a functional NS3/4A protease (wtNS3/4A and coNS3/4A) a cleavage of IPS-1 generates two distinct bands on the membrane. mNS3/4A has a mutation that prevents the release of NS4A and the generation of the complete NS3/4A complex (D). Comparison of the efficiency of human IPS-1 cleavage by the NS3/4A sequence with alanine mutations at residues 111, 112 or 113 of the NS3 protease with the cleavage of murine IPS-1 (E). Also shown is a parallel analysis of the cleavage of mouse IPS-1 by HBcAg (negative control), wild-type NS3, mutant NS3/4A with an impaired protease, and coNS3/4A with a functional protease. All experiments shown have been repeated two to five times.

We wanted to emulate the effects of an infection with an RNA virus without activating the specific immune response, since this would complicate analysis of innate responses in vivo. We therefore used synthetic dsRNA, or poly(I:C), which only activates the innate responses. After intravenous injection of poly(I:C) the nuclear translocation of IRF-3 and NF-κB by the RIG-I/TLR3-dependent pathway was determined (fig 2A). As a comparison, the NS3/4A-Tg mice were also treated with TNFα/d-GalN (fig 2B), representing a RIG-I/TLR3-independent pathway.^33^ A reduced nuclear translocation of IRF-3 and NF-κΒ in the NS3/4A-Tg mice showed that the response to dsRNA was impaired in vivo (fig 2A). In contrast, treatment with TNFα/d-GalN had no effect on IRF-3 (fig 2B), whereas the NF-κB levels were increased in the NS3/4A-Tg mice (fig 2B). The latter is now a well-documented finding in these mice (Weiland *et al*, manuscript in preparation). Thus, expression of NS3/4A in mouse livers, at around 25-fold lower levels as compared to those seen in HCV infected humans, specifically inhibits the RIG-I/TLR3-dependent activation of IRF-3 and NF-κB by dsRNA. Thus, NS3/4A modulates the innate murine response to dsRNA in vivo.

### NS3/4A cleaves human and murine IPS-1

The best-documented effects of NS3/4A on cell signalling is the cleavage of human IPS-1 from the mitochondrial membrane.^19^ ^23^ ^34^ Whether the same is true for IPS-1 of other species, except for tamarins,^35^ is not known. We first tested the cleavage of murine IPS-1 by an in vitro transcription and translation assay (fig 2C). Co-translation of mIPS-1 with NS3/4A results in a reduction of free NS3, and the appearance of a band consistent with truncated (Δ) IPS-1 (fig 2C and data not shown). Hence, the cleavage of murine IPS-1 is competing with the cleavage at the NS3–NS4A junction resulting in a reduction of free NS3. This was confirmed by over-expressing human and murine IPS-1 in HepG2 cells, where co-transfection with NS3/4A resulted in an extra band representing ΔIPS-1 (fig 2D). Thus, HCV NS3/4A cleaves both human and murine IPS-1. The exact position for cleavage of murine IPS-1 is under investigation, although the predicted site at the cysteine residue at position 470 of the mouse IPS-1 should be a good candidate.^35^ To better characterise the difference in the effectiveness of cleavage of the human and mouse IPS-1 we co-transfected HepG2 cells with versions of our wild-type gt1a NS3/4A sequence having single alanine substitutions and with intact protease activity.^13^ ^14^ Using these mutant NS3/4A sequences we did see differences in the efficiency of cleaving of the human IPS-1 gene (fig 2E), suggesting that the generally fainter ΔmIPS-1 band observed is a result of a less efficient cleavages as compared to human IPS-1 (fig 2E).

### NS3/4A does not protect transiently transfected hepatocytes, as a model of infected hepatocytes, from elimination by specific T cells

**Figure 3:**
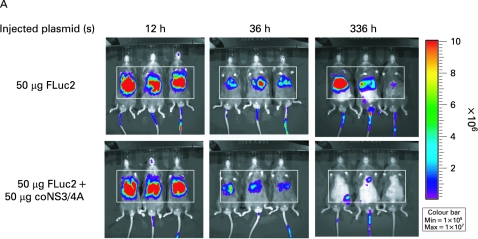

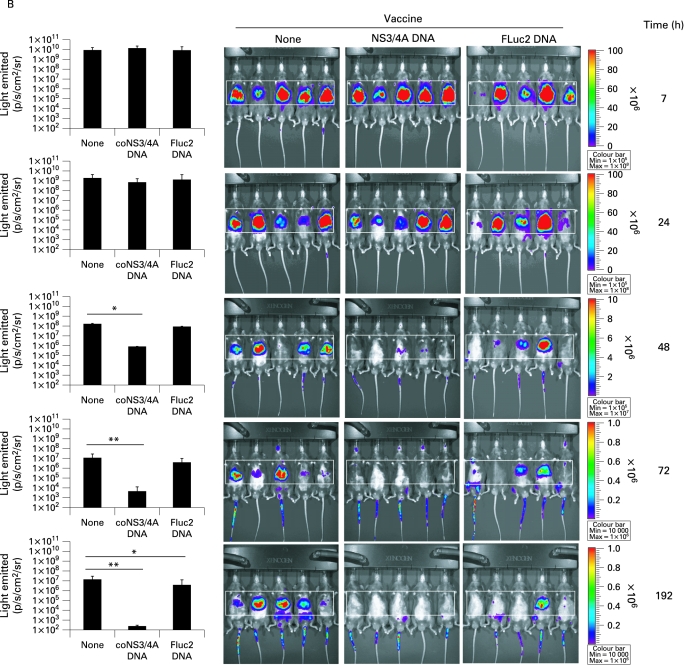
The non-structural (NS) 3/4A complex does not promote survival of firefly luciferase (FLuc) expression in co-transfected hepatocytes in vivo. Real-time in vivo imaging of FLuc expression in mice 12, 36 and 336 h after a hydrodynamic injection of FLuc alone or in combination with coNS3/4A (A) NS3/4A does not prevent clearance of NS3/4A-FLuc co-transfected hepatocytes in mice intramuscularly immunised with phosphate-buffered saline (PBS), coNS3/4A-pVAX1 or FLuc-pVAX1 2 weeks prior to hydrodynamic injection (B). (See p 565 for panel B.) The presence of a statistical difference has been indicated as follows: *p<0.05; **p<0.01, Mann–Whitney U test. The area used for calculation of light intensities has been boxed. All experiments shown have been repeated at least thrice.

Since NS3/4A inhibits the innate murine immune response, an immediate question is whether this also has any clear effects on the adaptive hepatic immune responses. We therefore used in vivo imaging to study the real-time kinetics of clearance of hepatocytes co-expressing NS3/4A and FLuc generated by a hydrodynamic injection. Co-expression of NS3/4A did not promote survival of FLuc-expressing cells in naive mice, where hepatocytes co-expressing NS3/4A and FLuc persisted for fewer than 14 days (336 h; fig 3A) but more than 8 days (192 h; fig 3B). In contrast, mice with pre-existing NS3/4A-specific CTLs had cleared most NS3/4A-FLuc co-expressing hepatocytes between 24 and 48 h (fig 3B). Collectively, NS3/4A cannot protect transfected, or “infected”, hepatocytes from clearance by specific T cells. This is consistent with the idea that IPS-1 does not have a major influence on the adaptive immunity.^24^ ^25^

### Clearance of NS3/4A-expressing hepatocytes is dependent on classical molecules involved in the killing of virus-infected cells

To define molecules involved in the effector-mediated clearance of NS3/4A-protein expressing hepatocytes, we studied hepatic entry of CD3+ cells and elimination of hepatic NS3/4A-protein expression in vaccinated gene deficient “knock-out” mice. The role of the molecules was defined by a hydrodynamic challenge with an NS3/4A-expressing plasmid,^30^ in naive or vaccinated mice lacking specific genes involved in T cell priming or effector function (CD4 and CD8), T cell trafficking (TLR4 and IFNγ receptor-2),^36^ and lytic killing of virally infected cells (perforin and FasL).^37^ ^38^

**Figure 4:**
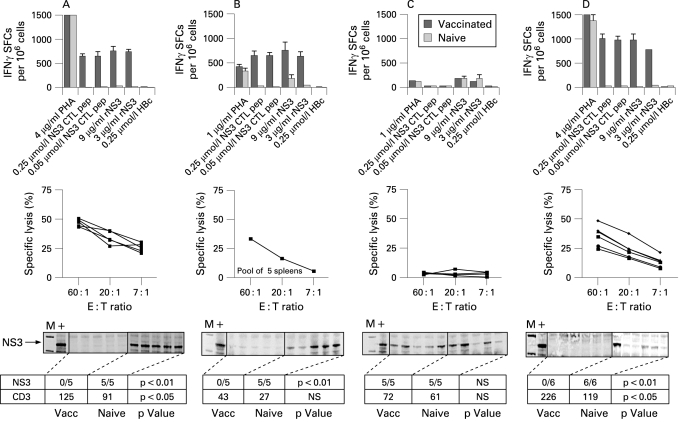

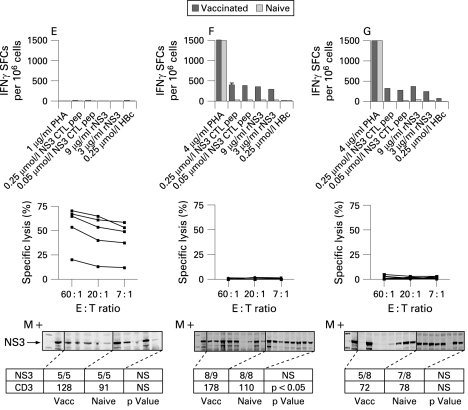
Presence of non-structural (NS)3-specific interferon γ (IFNγ)-producing spot forming cells (SFCs) by ELISpot (bar graphs), lytic cytoxic T cells (CTLs; line graphs), and hepatic NS3 (gels) in vaccinated and non-vaccinated mice receiving a hydrodynamic challenge. SFCs were counted after stimulation with a H-2D^b^ restricted major histocompatibility complex (MHC) class I peptide (GAVQNEVTL), recombinant (r) NS3, or an irrelevant MHC class I peptide (HBc) in pooled lymph nodes from naive or gene-gun immunised wild-type (A), CD4−/− (B), CD8−/− (C), TLR4−/− (D), IFNγR2−/− (E), perforin−/− (F) and FasL−/− (G) mice. (See p 567 for panels E to G.) A cut-off at 50 SFCs/10^6^ cells indicates a positive IFNγ response. NS3-specific lytic CTL activity using peptide was determined using peptide-loaded RMA-S cells in splenocytes from individual mice, re-stimulated in the presence of the H-2D^b^ restricted MHC class I peptide. Specific lysis was determined at effector:target ratios of 60:1, 20:1 and 7:1 in a standard ^51^Cr-release assay. Cut-off for a positive lysis has been set to 20% specific lysis. In vivo clearance of transient hepatic NS3/4A-protein expression was analysed by immunoprecipitation and western blot 72 h after the hydrodynamic injection. “M” indicates the molecular rainbow marker. “+”, positive control (eg, coNS3/4A transiently transfected baby hamster kidney-21 (BHK) cells). The number of NS3-protein positive mice per group is indicated in the row below each gel. p Values indicate a statistical difference (p<0.05 or p<0.01) between naive and vaccinated mice as determined by Fisher’s exact test (Statview). In the second row below the gel, the mean number of detectable infiltrating CD3+ T cells/10 mm^2^ in the livers of groups of mice has been given. p Values indicate a statistical difference (p<0.05 or p<0.01) between naive and vaccinated mice as determined by the Mann–Whitney U test (Statview). Experiments that had been run previously (wild-type, CD4−/−, CD8−/− and IFNγR2−/−) were run once with five mice per group, whereas the other experiments were run with a higher number of animals per group (n = 6–9). Since results were consistent the latter were only run once.

In wild-type mice, an NS3/4A-DNA vaccination activates IFNγ-producing Th cells and CTLs, and the CTLs have lytic activity in vitro (fig 4A). These T cells enter the liver, evidenced by an increase in hepatic CD3+ cells, and are functional, since transiently NS3/4A-transgenic hepatocytes are cleared within 72 h (fig 4A). Consistent with previous reports,^26^ ^30^ were CD8+, but not CD4+ cells required for clearance (fig 4B,C). CD4−/−, but not CD8−/− mice developed lytic and IFNγ-producing T cells that entered the liver and eliminated NS3/4A-protein expressing hepatocytes (fig 4B,C). This is consistent with our previous observation that priming and clearance of NS3/4A-specific CTLs is independent of CD4+ cells, but dependent on CD8+ T cells.^26^ ^30^

Next, IFNγR2, but not TLR4, was required for clearance of NS3/4A-protein expressing hepatocytes (fig 4D,E), even though the IFNγR2−/− mice had a strong lytic activity in vitro (fig 4E). This may possibly be explained by the fact that the CTLs failed to enter the liver (fig 4E), consistent with recent data showing that IFNγ is involved in trafficking of T cells to infected sites.^36^ However, we found no role for TLR4 in either priming or hepatic recruitment of newly primed NS3/4A-specific CTLs (fig 4D), which is different from the suggested role of TLR4 in trapping memory cells in the liver.^39^

Lastly, hepatic clearance of NS3/4A-protein-expressing cells was dependent on classical molecules involved in killing of virally infected cells. Mice lacking perforin or FasL had a significantly impaired clearance of hepatic NS3/4A-protein expression (fig 4F,G), strongly suggesting that these molecules are required for clearance of these cells. Both perforin−/− and FasL−/− mice failed to develop lytic T cells that were detectable in vitro, although IFNγ-producing cells were recalled in vitro confirming that T cells had been primed (fig 4F,G). Also, the vaccinated perforin−/− mice had an increased frequency of CD3+ cells in the liver (fig 4F), suggesting that the absence of perforin affects killing rather than hepatic trafficking of T cells. Importantly, these data also suggest that NS3/4A does not actively interfere with killing mechanisms exerted by perforin and Fas. In conclusion, using transiently NS3/4A Tg mice with hepatic NS3/4A expression at levels comparable to humans, we found no apparent role for NS3/4A to participate in the observed inhibition of CTL responses observed in HCV-infected individuals^10^ and transgenic mice.^15^

## DISCUSSION

The underlying mechanisms that help HCV to establish and maintain a chronic infection in humans are still poorly understood.^4^ ^11^ ^40^ Several mechanisms that seem to promote viral persistence have been identified in vitro and in animal studies.^7^ ^41^^–^44 Many HCV proteins seem to interfere with host cell signalling, where the NS3/4A complex is the best characterised, so far.^3^ ^19^ ^20^ The NS3/4A-mediated cleavage of IPS-1 has been documented in human and tamarin cells.^34^ ^35^ However, there are significant difficulties in studying these events repeatedly in infected human livers. It is therefore of the utmost importance to determine if there are other simpler model systems that may in the same, or in a different way, represent the in vivo situation. Since a number of transgenic mouse lineages exist where NS3/4A should be present,^16^ ^17^ ^45^ we studied the effects of NS3/4A in murine cells.

In line with previous reports^3^ ^19^ ^23^ we found that over-expression of NS3/4A in human cells reduced poly(I:C)-induced IFN and NF-κB activation. One peculiar observation was that the reported cleavage of Toll-IL1 receptor domain containing adaptor inducing interferon β (TRIF) found in human cells,^20^ could not be documented in murine cells (unpublished observations) and this cleavage seems to differ between different human cell lines.^46^ The mice with stable transgene expression of NS3/4A had a lower expression of NS3/4A as compared to infected humans, whereas the levels in the transiently NS3/4A Tg mice were somewhat better comparable to humans. Thus, this suggests that transgenic mouse models are valid but these differences in expression levels need to be kept in mind. In addition, when NS3/4A is present in murine cells it seems to behave similarly as when present in human cells. This was supported by the observation that the NF-κB response to poly(I:C) was reduced in murine SP2/0 cells transiently transfected with NS3/4A. In addition, in poly(I:C)-treated NS3/4A Tg mouse livers the activation of IRF-3 and NF-κB was reduced, whereas, as predicted, TNFα failed to do the same.^33^ Thus, the effects seen on IRF-3 and NF-κB after poly(I:C) treatment were specific for the RIG-I/TLR3-dependent pathway, suggesting that the response to dsRNA was impaired by NS3/4A also in murine cells. We therefore tested whether our NS3/4A protein cleaved murine IPS-1. This was indeed the case as determined by both an in vitro transcription and translation assay as well as in transiently transfected cells. Comparison with the efficiency by which mutant NS3/4A sequences cleaved human IPS-1, suggested that the cleavage of mouse IPS-1 was less efficient than human IPS-1. Also, this suggests that different HCV NS3/4A sequences may cleave IPS-1 with different efficiencies, which certainly merits further investigation. Thus, it is likely to assume that the NS3/4A-mediated cleavage of murine IPS-1 is the cause for a reduced response to dsRNA in vitro and in vivo. This supports the findings from human tumour cells where NS3/4A blocks the dsRNA-induced interferon response mediated by RIG-I, IPS-1 and IRF-3.^3^ ^19^ ^23^ Hence, these signalling events may now be studied in detail in vivo, albeit with care, using mouse models.

A role for NS3/4A in blocking responses to dsRNA in viral pathogenesis seems obvious, since this should prevent induction of an antiviral state and apoptosis of infected cells. However, it is not known whether this extends to the adaptive immunity. There are several potential ways in which NS3/4A could inhibit the adaptive immunity directly or indirectly. For example, the cleavage of IPS-1 may inhibit chemokine release, hamper the recruitment of T cells to the liver, or inactivate various immune targets by blocking MHC class I presentation, or by preventing Fas or perforin/granzyme mediated killing. If this was indeed the case, this should be detectable in mouse livers transiently expressing NS3/4A as an impaired clearance of transfected cells. We could show that NS3/4A did not promote persistence of a co-transfected reporter gene by in vivo imaging. Also, we could show that the clearance of NS3/4A-protein expressing hepatocytes followed the classical pattern of lytic elimination requiring IFNγ, perforin and Fas, generally seen in viral infections.^37^ ^38^ ^47^ Thus, this strongly suggests that these pathways are not affected by NS3/4A. This issue was partially addressed also in a recent study using another RNA virus, the murine lymphocytoid choriomeningitis virus,^24^ showing that the priming of virus-specific CTLs by the infecting virus was independent of IPS-1. Taken together, this suggests that IPS-1 does not seem to be critical for CTLs either at the priming or the effector stage. Moreover, these data confirm our previous observation, that the reported inactivation of the Fas-related molecule Bid seen in mice that express the full HCV polyprotein,^15^ is probably not mediated by NS3/4A.^17^

In conclusion, the inhibition of dsRNA-induced RIG-I signalling was impaired in vivo in mice with 25-fold lower expression levels of NS3/4A than those seen in infected human livers. However, even when using mice with higher expression levels of NS3/4A, we found no clear evidence for an NS3/4A-mediated protective effect of “infected” cells towards CTLs. This is in line with recent studies, using unrelated RNA viruses, showing that IPS-1 is not required for the adaptive immune response.^24^ ^25^
